# Preliminary evidence of long‐term safety and effectiveness of anti‐CGRP drugs for migraine prevention in people with comorbid epilepsy: A 52‐week follow‐up retrospective study

**DOI:** 10.1002/epi4.70364

**Published:** 2026-10-09

**Authors:** Marina Romozzi, Andrea Burgalassi, Francesco Ceriello, Pietro Martino, Giuliano Sette, Francesca Pistoia, Fabio Placidi, Diego Centonze, Luca Pani, Jacopo Canonichesi, Catello Vollono, Paolo Calabresi, Maria Albanese, Luigi Francesco Iannone

**Affiliations:** ^1^ Dipartimento Universitario di Neuroscienze Università Cattolica del Sacro Cuore Rome Italy; ^2^ Neurologia, Dipartimento di Neuroscienze, Organi di Senso e Torace Fondazione Policlinico Universitario Agostino Gemelli IRCCS Rome Italy; ^3^ Headache Center and Clinical Pharmacology Unit AOU Careggi Florence Italy; ^4^ Department of Neuroscience, Mental Health and Sensory Organs (NESMOS) Sapienza University of Rome Rome Italy; ^5^ Neurology Unit Sant'andrea University Hospital Rome Italy; ^6^ Department of Biotechnological and Applied Clinical Sciences University of L'aquila L'Aquila Italy; ^7^ Neurology Unit, Department of Systems Medicine Tor Vergata University Rome Italy; ^8^ IRCCS Neuromed Pozzilli Italy; ^9^ Department of Biomedical, Metabolic and Neural Sciences University of Modena and Reggio Emilia Modena Italy; ^10^ Department of Psychiatry and Behavioral Sciences University of Miami Miami Florida USA; ^11^ Università Telematica San Raffaele di Roma Rome Italy; ^12^ San Raffaele Sulmona L'Aquila Italy

**Keywords:** anti‐CGRP drugs, CGRP, epilepsy, migraine, real‐world evidence

## Abstract

Migraine and epilepsy frequently coexist, but evidence on the safety of calcitonin gene‐related peptide (CGRP)‐targeting therapies in people with epilepsy is lacking. We evaluated long‐term migraine and epilepsy outcomes in a real‐world multicenter retrospective cohort of adults with comorbid migraine and epilepsy treated with anti‐CGRP monoclonal antibodies or gepants while on stable antiseizure medications. Eleven patients were included in the final analysis, all female, with a mean age of 34.7 ± 12.2 years. At baseline, mean monthly headache days (MHDs) were 18.5 ± 7.8, with 54.5% episodic migraine and 45.5% chronic migraine; 72.7% of patients had drug‐resistant epilepsy, and baseline mean monthly seizure frequency was 0.75. Mean MHDs decreased to 7.6 ± 5.5 at 24 weeks and to 5.4 ± 3.2 at 52 weeks (*p* < 0.001). At week 24, 90% of patients achieved a ≥50% reduction in MHDs, which was maintained at week 52; ≥75% response increased from 10% at week 24 to 20% at week 52, and 10% achieved migraine freedom at 1 year; MIDAS and HIT6 scores decreased significantly from baseline to both 24 and 52 weeks (all *p* < 0.001). Acute medication days and intake were reduced by over 85% at the end of the study. Seizure frequency did not increase during follow‐up and showed a non‐significant numerical reduction to 0.40 monthly seizures at 52 weeks (*p* = 0.423). No seizure exacerbations or treatment‐related adverse events were observed. This study provides preliminary, long‐term observations suggesting that anti‐CGRP therapies may be effective for migraine prevention in patients with comorbid epilepsy. No adverse events or apparent worsening of seizure frequency were observed during the 52‐week follow‐up. These findings require confirmation in larger, prospective controlled studies.

**Plain Language Summary:**

Migraine and epilepsy often occur together, but information on CGRP‐targeting migraine medicines in people with epilepsy is limited. We reviewed records from 11 women with both conditions over 1 year. Headache, migraine‐related disability, and use of medicines for attacks decreased. Among 10 women with complete migraine follow‐up, nine had at least half as many headache days at both 6 months and 1 year. No worsening of seizures or treatment‐related side effects was reported. These findings are encouraging, but this small study without a comparison group cannot establish safety or prove treatment benefit.

**Key points**

- A multicenter retrospective study followed 11 women with migraine and epilepsy receiving CGRP‐targeting therapies.
- Migraine frequency, disability, and acute medication use improved over 52 weeks.
- Nine of 10 patients with complete migraine follow‐up achieved a ≥50% reduction in monthly headache days at weeks 24 and 52.
- No seizure exacerbations or treatment‐related adverse events were observed; seizure frequency did not change significantly.
- The small, uncontrolled cohort provides preliminary findings that require confirmation in larger prospective studies.

## 1 INTRODUCTION

Migraine and epilepsy are among the most prevalent and disabling neurological disorders and were among the 10 neurological conditions contributing most substantially to global loss of health.^1^ Migraine affects approximately 14‐15% of the global population, while an estimated 0.7% of the population had active epilepsy in 2021.^2^, ^3^

These disorders are both characterized by recurrent, transient, and paroxysmal disturbances of the nervous system.^4^ Beyond their individual clinical impact, these conditions are closely related, sharing genetic susceptibility, pathophysiological mechanisms, overlapping symptomatology, and partially convergent therapeutic approaches.^5^ Epidemiological data consistently demonstrate a bidirectional association, whereby individuals affected by one disorder exhibit an increased risk of developing the other.^5^

This close relationship is also evident at the therapeutic level. Several antiseizure medications (ASMs) are used as migraine preventive treatments in patients with comorbid epilepsy.^6^, ^7^ However, their use is limited by relevant safety concerns, particularly in women of childbearing age, as well as tolerability issues (i.e., calcium channel blockers and beta‐blockers can induce or exacerbate somnolence or depression).^8^ Lastly, tricyclic antidepressants are associated with a potential, although low, risk of lowering the seizure threshold.^8^

Calcitonin gene‐related peptide (CGRP) may represent a key molecular link between migraine and epilepsy. CGRP is a neuropeptide with potent vasodilatory and neuromodulator properties.^9^ Its role in migraine pathophysiology has been extensively characterized, leading to the clinical development of monoclonal antibodies targeting CGRP or its receptor (anti‐CGRP mAbs) and small‐molecule CGRP receptor antagonists (gepants), which have significantly improved the efficacy and tolerability of migraine treatment.^10^

Emerging experimental evidence implicates CGRP also in epileptogenesis. CGRP is upregulated in the brain after sustained seizures and can induce epileptiform activity, neuroinflammation, and blood–brain barrier (BBB) disruption, although it may also exert neuroprotective effects during post‐injury recovery.^11^, ^12^ This duality suggests a context‐dependent role for CGRP in seizure‐related brain pathology.^13^

These observations raise the critical question of whether modulation of the CGRP pathway may influence epilepsy‐related outcomes, either adversely or beneficially. Preliminary observational data indicate that patients with comorbid migraine and epilepsy treated with CGRP inhibitors may experience fewer episodes of status epilepticus (SE), although clinical evidence remains limited. Given the high prevalence of migraine in people with epilepsy (PwE), the proven efficacy of anti‐CGRP drugs in migraine prevention, their favorable safety and tolerability profile, and the absence of known pharmacokinetic interactions with ASMs (especially anti‐CGRP mAbs), evaluating their real‐world use in PwE represents an important unmet clinical need.^14^

## 2 METHODS

### 2.1 Study design and population

This preliminary retrospective multicenter study included adults (≥18 years) with epilepsy (ILAE criteria) and migraine (ICHD‐3) treated with anti‐CGRP drugs for ≥6 months across four Italian referral centers. Patients were on stable ASM therapy at anti‐CGRP initiation. Those with incomplete data or changes in migraine preventives were excluded. Adverse events (AEs) were recorded.

#### 2.1.1 Variables collected

Collected migraine data included diagnosis, disease duration and onset, aura, anti‐CGRP treatment type and start date, concomitant preventives, monthly headache days (MHDs), numbers of analgesics (AMNs) and days with use of analgesics (AMDs), HIT‐6, and MIDAS. Outcomes (MHDs, AMNs, AMDs, HIT‐6, MIDAS) were assessed at baseline (T0), week 24 (T1), and week 52 (T2).

Epilepsy data included duration, type, etiology, baseline monthly seizure frequency (3 months pre‐T0), drug resistance (ILAE), time since last seizure, and ASM regimens at T0–T2, including changes and reasons. The study was approved by the Ethics Committee of Università Cattolica del Sacro Cuore (ID 26959).

#### 2.1.2 Outcomes and safety monitoring

The outcomes were: (i) the change in MHDs, AMNs, AMDs, HIT‐6, MIDAS from baseline (T0) at week 24 and 52, and (ii) the proportion of responders (reduction of MHDs ≥50% [RR50%] compared to T0) at week 24 (T1) and 52 (T2).

For epilepsy, outcome measures included change in monthly seizure frequency from baseline (T0) to T1 and T2 after initiation of anti‐CGRP drugs, ASMs modification, and the occurrence of epilepsy‐related AEs.

### 2.2 Statistical analysis and missing data

Continuous variables were reported as mean ± standard deviation (SD) or median, interquartile range (IQR), and categorical data as numbers and percentages. Longitudinal changes in clinical variables at baseline (T0) and follow‐up time points (T1 and T2) were analyzed using the Friedman test. When a significant overall effect was observed, post hoc pairwise comparisons were performed using the Durbin‐Conover test. Comparisons between two paired time points were conducted using the Wilcoxon signed‐rank test when applicable. A two‐sided *p*‐value <0.05 was considered statistically significant. All data were analyzed using SPSS software version 29.0, and graphs were designed using GraphPad Prism version 10.00.

## 3 RESULTS

A total of 12 patients with comorbid migraine and epilepsy were identified, and 11 were included and analyzed in the study throughout 52 weeks (one patient was excluded due to incomplete data on seizure outcomes) (the flowchart is shown in Figure 1). All the demographic and clinical characteristics are reported in Table 1.

**FIGURE 1:**
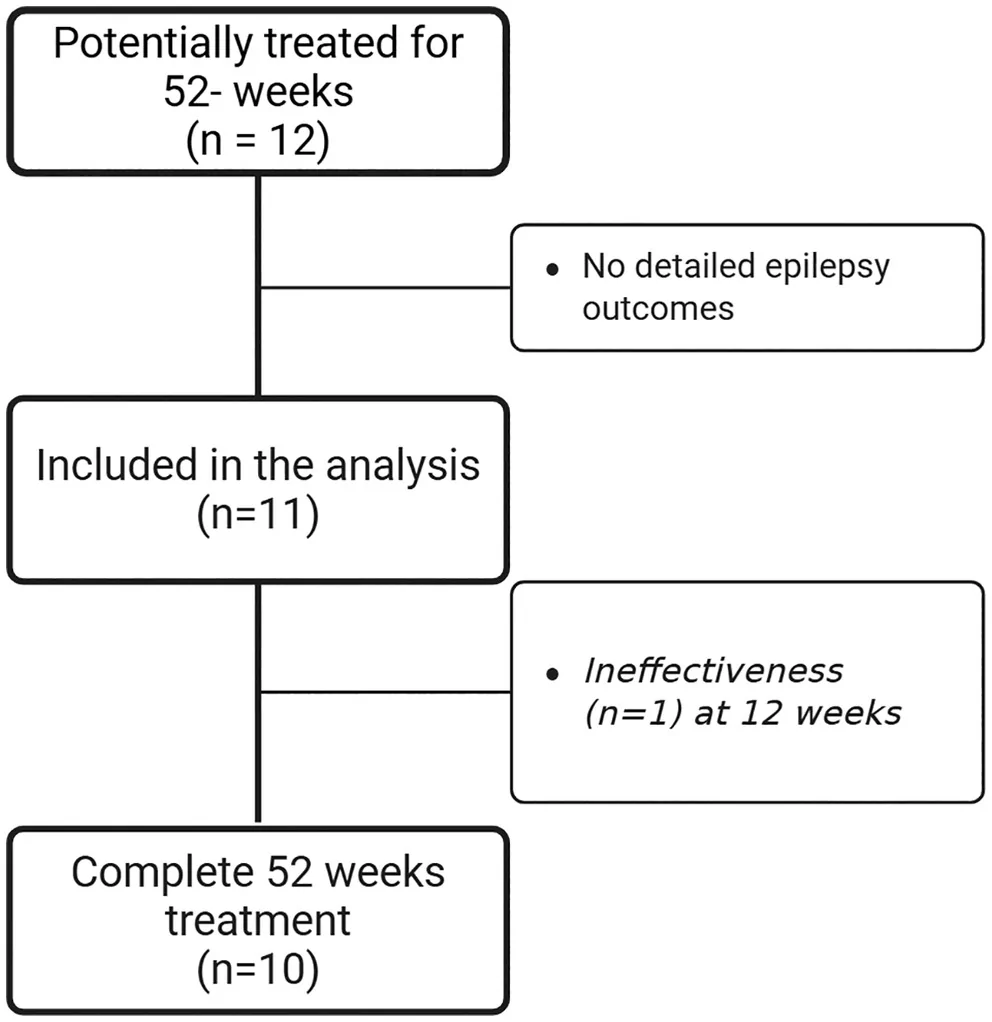
Flow diagram of people with epilepsy treated with anti‐CGRP drugs.

**TABLE 1:** Clinical and demographic data of people with migraine and comorbid epilepsy at baseline.

Patient	Sex	Age (years)	Comorbidities	MHDs	Migraine type	MOH	Aura	Migraine/epilepsy duration (years)	AMNs	AMDs	HIT‐6	MIDAS	Anti‐CGRP pathway treatment	Epilepsy type (etiology)	DRE	ASMs
1	F	25	‐	14	EM	Yes	No	8/6	14	14	69	34	FREMA	IGE	No	TPM
2	F	30	‐	25	CM	No	No	1/20	5	5	72	23	FREMA	Focal (unknown)	Yes	LEV
3	F	43	‐	16	EM	No	No	20/20	7	7	68	95	FREMA	Focal (Structural)	Yes	PB
4	F	35	Microcytemia; nephrolithiasis	14	EM	No	Yes	15/6	2	2	66	35	ATO	Focal (Structural)	Yes	LEV
5	F	30	Anxiety	18	CM	Yes	Yes	10/8	15	15	70	40	ATO	Focal (Structural)	Yes	LCM
6	F	27	‐	8	EM	No	No	14/16	8	8	68	14	GALCA	Focal (Structural)	Yes	BRV, CBZ
7	F	61	Insomnia	30	CM	Yes	No	50/45	120	30	60	59	FREMA	IGE	No	VPA
8	F	50	Asthma; Insomnia; Celiac disease	30	CM	No	No	14/20	10	10	65	57	ERE	IGE	Yes	VPA, CLN
9	F	22	PFO; Hypotension.	11	EM	No	No	4/11	5	5	62	42	FREMA	IGE	Yes	LEV, TPM
10	F	36	Cerebral hemorrhage; Mood disorder	25	CM	No	No	15/25	2	2	66	35	FREMA	Focal (Structural)	Yes	CLN
11	F	23	Depression; Thyroid nodule	12	EM	No	No	12/13	10	10	63	38	FREMA	IGE	No	TPM

Abbreviations: AMNs, Number of medications per months; ASMs, Anti‐seizure medications; ATO, atogepant; BRV, Brivaracetam; CBZ, Carbamazepine; CLN, Clonazepam; CM, Chronic migraine; DRE, drug‐resistant epilepsy; EM, Episodic migraine; ERE, erenumab; F, Female; FREMA, fremanezumab; GALCA, galcanezumab; HIT‐6, Headache Impact Test‐6; IGE, Idiopathic generalized epilepsy; LCM, Lacosamide; LEV, Levetiracetam; MHDs, Monthly headache days; MIDAS, Migraine Disability Assessment; MMD, Monthly migraine days; MOH, Medication‐overuse headache; n/a, Not available; PB, Phenobarbital; PFO, Patent foramen ovale; TPM, Topiramate; VPA, Valproic acid.

At the time of anti‐CGRP drug initiation, eight patients (72.7%) had drug‐resistant epilepsy and were seizure‐free for a mean (SD) duration of 37.0 (51.3) months. One patient (9.1%) fulfilled the criteria for migraine aura‐triggered seizure, and two patients (18.2%) fulfilled the ICHD‐3 criteria for post‐ictal headache.

### 3.1 Impact on migraine‐related outcomes

After discontinuation for lack of effectiveness in one patient at week 12, 10 patients had complete data at week 24 (T1) and 52 (T2).

There was a significant reduction of MHDs across the three assessment points T0, T1, and T2 (*p* < 0.001), with a significant reduction in MHD at T1 (mean difference = 11.5, *p* < 0.001) and at T2 (mean difference = 13.70, *p* < 0.001) compared with baseline (T0) and between T1 and T2 (mean difference = 2.20, *p* = 0.020) (Figure 2A).

**FIGURE 2:**
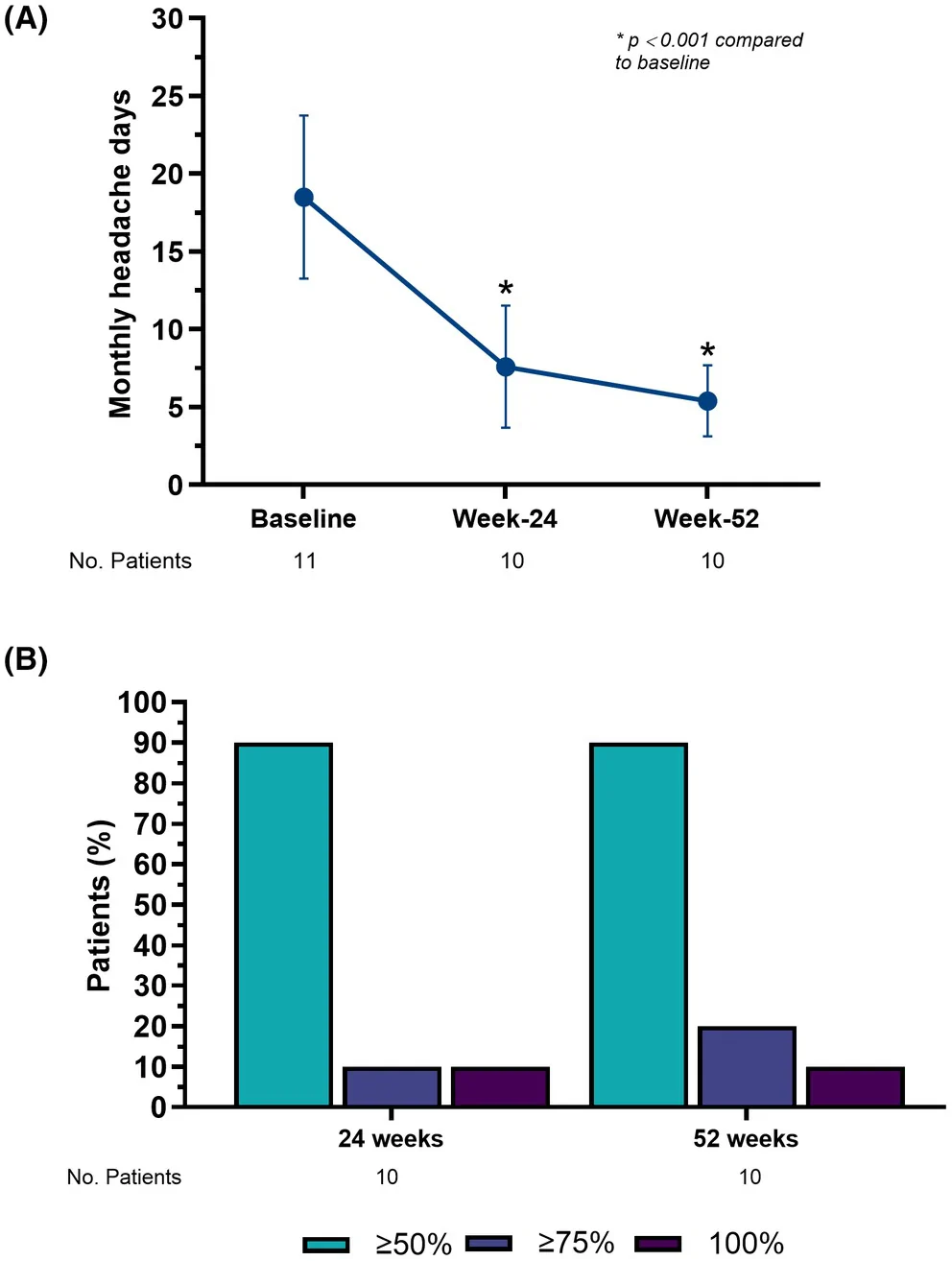
Monthly headache days reduction (A) and response rates (B) at week 24 and week 52. Percentages reported are cumulative.

Regarding disability, the HIT‐6 and MIDAS scores were significantly different among the three time points (*p* < 0.001), with significant reduction in MIDAS and HIT‐6 at T1 (mean difference = 12.60, *p* = 0.002 and 27.20, *p* = 0.018) and at T2 (mean difference = 14.00, *p* < 0.001 and 37.0, *p* <0.001) compared with T0, respectively.

A significant decrease in AMDs was shown across the three assessment points (*p* < 0.001). Post hoc pairwise comparisons showed a significant difference from T0 to T1 (mean difference = 8.9, *p* < 0.001), T0 and T2 (mean difference = 7.5, *p* < 0.001), and from T1 to T2 (mean difference = 1.4, *p* = 0.020).

Similarly, AMNs decreased significantly across the three assessment points (*p* < 0.001) (Table 2).

**TABLE 2:** Migraine baseline features and treatment outcomes at 24 and 52 weeks for the cohort included.

					Post‐hoc analyses		
*(Mean ± SD)*	Baseline (T0) (*n* = 11)	Week 24 (T1) (*n* = 10)	Week 52 (T2) (*n* = 10)	*p*‐value	T1 vs. T0	T2 vs. T0	T2 vs. T1
MHDs	18.5 ± 7.8	7.6 ± 5.5	5.4 ± 3.2	<0.001	<0.001	<0.001	0.020
HIT‐6	66.3 ± 3.6	54.0 ± 9.04	52.6 ± 8.06	<0.001	<0.001	<0.001	0.001
MIDAS	42.9 ± 21.512	16.2 ± 18.0	6.4 ± 4.8	<0.001	<0.001	<0.001	0.015
AMNs	18.0 ± 34.1	2.3 ± 2.3	0.9 ± 1.7	<0.001	<0.001	<0.001	0.020
AMDs	9.8 ± 7.9	2.3 ± 2.3	0.9 ± 1.7	<0.001	<0.001	<0.001	0.020

Abbreviations: AMNs, Number of medications per month; HIT‐6, Headache Impact Test‐6; MHDs, Monthly headache days; MIDAS, Migraine Disability Assessment; MMD, Monthly migraine days; SD, standard deviation.

At 24 weeks (T1), 9/10 patients (90%) achieved a ≥50% reduction in monthly headache days (responders), with 10% achieving ≥75% and 10% complete freedom. At 52 weeks (T2), 90% maintained responder status; ≥75% reduction increased to 20%, and 10% remained migraine‐free (Figure 2B).

### 3.2 Impact on epilepsy related outcomes

During anti‐CGRP treatment, there was no significant change in seizure frequency across the three assessment points (*p* = 0.670). The mean monthly seizure frequency decreased from 0.75 at baseline (T0) to 0.40 at 52 weeks of anti‐CGRP treatment (*p* = 0.423).

### 3.3 Concomitant treatments and safety

Regarding ASMs modifications during treatment with anti‐CGRP drugs, one patient with two ASMs at baseline (levetiracetam and topiramate) suspended topiramate. No patient experienced seizure exacerbation, serious AEs, or any drug‐related AE.

One patient discontinued an anti‐CGRP mAb (fremanezumab) at month 3 for lack of effectiveness, according to clinical judgment.

## 4 DISCUSSION

In this small retrospective cohort of people with migraine and comorbid epilepsy, anti‐CGRP pathway treatment was associated with improvements in migraine frequency, migraine‐related disability, and analgesic use over 52 weeks. No adverse events or apparent increase in seizure frequency were observed. Given the small sample size and study design, these findings should be considered preliminary.

The effectiveness observed in this cohort appears broadly consistent with outcomes reported in studies of anti‐CGRP therapies in the general migraine population.^15^ However, this indirect comparison should be interpreted cautiously because of differences in study design and patient characteristics and the absence of a migraine‐only control group.

Regarding epilepsy outcomes, anti‐CGRP therapy did not negatively affect seizure frequency over the 52‐week follow‐up; on the contrary, a numerical, although not statistically significant, reduction in seizure frequency was observed. No AEs were reported.

While there are no similar data in the literature, data from a large retrospective cohort study compared PwE who were on CGRP inhibitors against matched epilepsy patients not on anti‐CGRP drugs.^16^ The results showed that the group receiving anti‐CGRP therapy had a significantly lower reduction in occurrence and likelihood of SE. Furthermore, hospital visits for SE were significantly less frequent in patients on CGRP pathway inhibitors than in the control group.^16^

Experimental data indicate that CGRP participates in seizure‐related neuroinflammation and excitability. It is markedly upregulated after prolonged or injurious seizures, but not after brief, self‐limited events.^11^, ^12^ However, CGRP also has neuroprotective effects,^13^ suggesting a context‐dependent role in both seizure propagation and post‐seizure repair.

Although anti‐CGRP pathway drugs have minimal or negligible penetration of an intact BBB, they may still influence seizure outcomes.^17^ CGRP signaling is prominent in extracerebral sites such as the meninges and peripheral sensory ganglia, which connect to brainstem and thalamocortical circuits regulating excitability.^17^ Thus, peripheral CGRP blockade may indirectly modulate central network excitability.

Regarding safety and tolerability, anti‐CGRP therapies represent a valid option owing to their neutral and potentially beneficial effects on psychiatric comorbidities and sleep disturbances, which are particularly prevalent in PwE^18^ Importantly, these drugs do not cause systemic sedation, cognitive slowing, or organ toxicity, making them particularly attractive for use in PwE.

The study's strengths include 52‐week real‐world follow‐up and a well‐characterized cohort with severe, drug‐resistant epilepsy, often excluded from trials. Limitations include the retrospective design, small sample, and no control group, limiting generalizability. Cohort heterogeneity and small subgroups prevented analyses by epilepsy type, etiology, seizure burden, or treatment class.

## AUTHOR CONTRIBUTIONS

M.R. and L.F.I. contributed to conceptualization. M.R. and L.F.I. contributed to methodology. M.R., L.F.I., P.M., F.C., F.P., A.B., and M.A. performed the investigation and data curation. M.R. conducted the formal analysis. M.R. prepared the figures and tables. M.R. and L.F.I. coordinated the study, and F.P., D.C., L.P., P.C., and C.V. provided scientific supervision. M.R. and L.F.I. wrote the original draft. All authors critically reviewed and edited the manuscript, read and approved the final version, and agreed to be accountable for all aspects of the work, ensuring that questions related to its accuracy or integrity are appropriately investigated and resolved.

## FUNDING INFORMATION

This research did not receive any specific grants from funding agencies in the public, commercial, or not‐for‐profit sectors.

## CONFLICT OF INTEREST STATEMENT

M.R. received financial support, consulting fees for participation in advisory boards, and support for attending meetings from: Lundbeck, Pfizer, and AbbVie. L.F.I. received financial support, consulting fees for participation in advisory boards, and support for attending meetings from: Teva, Eli Lilly, Lundbeck, Pfizer, Organon, and AbbVie; he is Associate Editor for Frontiers in Neurology and junior editor for Confinia Cephalagica, Cephalalgia, and Cephalalgia Report. L.P. is/has been a consultant or advisory board member for: AbbVie, USA; BCG, Switzerland; Boehringer Ingelheim International GmbH, Germany; GH‐Research, Ireland; Immunogen, USA; Johnson & Johnson USA; LB Pharmaceuticals, USA;Magdalena BioSciences, USA; Sanofi‐Aventis‐Genzyme, France and USA; Lundbeck, Denmark and Italy; Napo‐Pharma, USA and EU; NetraMark, Canada; Pfizer Global, USA; Relmada Therapeutics, USA; Takeda, USA and owns shares/options from AiDvance Germany; Adapt UK, Enzeta USA, NetraMark Canada, and Relmada, USA. J.C. receives financial support from the Italian Ministry of Health (Ricerca Corrente 2026). Other authors have no relevant financial or non‐financial interests to disclose.

## ETHICS STATEMENT

We confirm that we have read the Journal's position on issues involved in ethical publication and affirm that this report is consistent with those guidelines.

## Data Availability

The data that support the findings of this study are available from the corresponding author upon reasonable request.
